# Pomelo peel oil suppresses TNF-α-induced necroptosis and cerebral ischaemia–reperfusion injury in a rat model of cardiac arrest

**DOI:** 10.1080/13880209.2021.1903046

**Published:** 2021-04-01

**Authors:** Wenyan Wang, Lu Xie, Xinsen Zou, Wanxiang Hu, Xinyue Tian, Gaoyang Zhao, Menghua Chen

**Affiliations:** a Intensive Care Unit, The Second Affiliated Hospital of Guangxi Medical University, Nanning, People’s Republic of China; b Department of Physiology, Guangxi Medical University, Nanning, People’s Republic of China

**Keywords:** *Citrus maxima*, cardiopulmonary resuscitation, mixed lineage kinase domain-like protein, receptor-interacting serine/threonine kinase 3, limonene, myrcene

## Abstract

**Context:**

Pomelo peel oil (PPO) [*Citrus maxima* (Burm.) Merr. (Rutaceae)] is reported to possess antioxidant and antimelanogenic activities.

**Objective:**

To investigate the effect of PPO [*Citrus maxima* (Burm.) Merr. *cv. Shatian Yu*] on tumour necrosis factor-α (TNF-α)-induced necroptosis in cerebral ischaemia–reperfusion injury (CIRI) after cardiac arrest (CA).

**Materials and methods:**

Male Sprague Dawley rats were randomly assigned to six groups: sham group, PP0-L (10 mg/kg), PPO-M (20 mg/kg), PPO-H (40 mg/kg) and two control groups (CA, 0.9% saline; Gly, 10% glycerol). All drugs were administered intravenously to the CA/CPR rats within 10 min after return of spontaneous circulation (ROSC). After 24 h, rats were assessed for neuronal injury via the neurological deficit score (NDS), cerebral cortex staining and transmission electron microscopy (TEM) and expression levels of TNF-α and necroptosis-related proteins by immunoreactivity staining and western blotting.

**Results:**

Compared to those in the sham group (survival rate, 100% and NDS, 80), the survival rate and NDS were significantly reduced in the model groups (CA, 56.25%, 70; Gly, 62.5%, 71; PPO-L, 75%, 72; PPO-M, 87.5%, 75; PPO-H, 81.25%, 74). In the PPO-M group, Nissl bodies were significantly increased (43.67 ± 1.906 vs. 17 ± 1.732), the incidence of pathomorphological injury was lower and the necroptosis markers (TNF-α, RIPK1, RIPK3, p-MLKL/MLKL) expression was downregulated compared to those in the CA group (*p* < 0.05).

**Discussion and conclusions:**

The neuroprotective effects of PPO in the CA rats suggested that PPO possibility as a health product enhances the resistance ability against brain injury for humans.

## Introduction

Cardiac arrest (CA) is the most critical clinical emergency and a major cause of death worldwide (Jentzer et al. 2016; Kelly and Pinto 2019). The high hospital mortality rate of patients who restore spontaneous circulation through cardiopulmonary resuscitation (CPR) is due to cerebral ischaemia–reperfusion injury (CIRI) (Choi et al. 2019). CIRI is damage to the brain’s structure and function, occurring when the blood supply returns to the brain tissues after cerebral ischaemia. Extensive brain damage is the leading cause of coma and death in patients who have undergone CA/CPR (Moore et al. 2017); therefore, it is vital to protect neurological function by preventing CIRI-induced cell death.

Necroptosis is a form of programmed necrosis mediated by receptor-interacting serine/threonine kinase 3 (RIPK3) and mixed lineage kinase domain-like protein (MLKL). It contributes to many types of acute tissue injury, such as stroke, myocardial infarction and ischaemia/reperfusion injury (Liu et al. 2019). Interestingly, pre-treatment with a specific necroptosis inhibitor, necrostatin-1 (Nec-1), ameliorates brain injury following middle cerebral artery occlusion (Li et al. 2019). However, the involvement of necroptosis in CIRI after CPR remains to be substantiated, and it is unclear if inhibiting necroptosis will exert neuroprotective effects after CA/CPR.

Pomelo [*Citrus maxima* (Burm.) Merr. (Rutaceae)] is one of the most popular fruit crops worldwide (Ding et al. 2013; Cui et al. 2018; Zhao et al. 2019). Gas chromatography–mass spectrometry (GC–MS) analysis of the chemical composition of fat solvent-extracted pomelo peel oil (essential) (PPO) identified limonene as the most abundant ingredient (55.92%), followed by β-myrcene (31.17%) and β-pinene (3.16%) (He et al. 2019). Additionally, the volatile oil extracted from fresh Taiwan Matou pomelo [*Citrus grandis* (L.) Osbeck *cv. Matou Wentan*] was analysed, and the main components were also identified as limonene (87.5%), myrcene (3.1%) and β-pinene (2.7%) (Chen et al. 2018). These molecules have antioxidant, anti-metabolic disorder and antiapoptotic properties (Chen et al. 2018; He et al. 2019). Therefore, we hypothesized that PPO may play a neuroprotective role after CA/CPR. In this study, we investigated the effects of PPO on tumour necrosis factor-α (TNF-α)-induced necroptosis in CIRI in a rat model of CA/CPR.

## Materials and methods

### Sample collection and extract preparation

Ripe pomelos [*Citrus maxima* (Burm.) Merr. *cv. Shatian Yu*] were freshly collected from Shatian village, Rong County, Guangxi Zhuang Autonomous Region, in mid-September of 2019. The specimen was identified by Professor Yusong Huang from Herbarium (IBK), Guangxi Institute of Botany, Guangxi Zhuang Autonomous region and Chinese Academy of Sciences, and the specimen (W.Y. Wang 201909001) was stored and catalogued in IBK.

The pulp and white skin of pomelos were removed after washing with purified water, and the peel was cut into pieces. The volatile oil was extracted from pomelo peel by steam distillation (Saviuc et al. 2010; Zou et al. 2018). Specifically, 150 g of chopped pomelo peel along with 200 mL of pure water were placed into a round bottom flask that was subsequently attached to a Soxhlet extraction device. During boiling, the essential oil of pomelo was condensed by a reflux condensation tube, then flowed to a side pipe (siphon) where PPO was separated from water. The extraction took 12 h. Pomelo peel (150 g) produced approximately 300 μL of PPO. We repeated the extraction to collect about 2 mL PPO and refrigerated at −4 °C for experimental preparation. Next, PPO was dissolved in 10% glycerine (v/v) for administration.

### Gas chromatography–mass spectrometry analysis of pomelo peel oil

PPO was qualitative analysed by GC (Shimadzu, GC-2010plus, Kyoto, Japan)–MS (Thermo Fisher, GC-MS TRACE1300, Waltham, MA). GC–MS was performed using a DB-5 fused quartz capillary column (30 m × 0.32 mm internal diameter, film thickness 0.1 µm; Agilent Technologies Inc., Santa Clara, CA). The heating program was set at an initial temperature of 50 °C. The heating program is maintained at 50 °C for 1 min, then heated up to 70 °C (at a rate of 3 °C/min) for 1 min, followed by 120 °C (at a heating rate of 3 °C/min) for 1 min, next, increased to 180 °C (at a rate of 5 °C/min) for 1 min and at last heated up to 250 °C (at a rate of 8 °C/min) for 1 min; finally, the temperature reached 320 °C at a rate of 15 °C/min until the analysis was complete. The carrier gas was helium, and the temperature of the injector and detector was set to 250 °C. The scanning range of the mass spectrometer was *m/z* 60–650 and the scanning speed was 4.0 bpm.

### Animals and experimental group

Healthy adult male Sprague Dawley rats (8 weeks old, 200–250 g; Experimental Animal Center of Guangxi Medical University, Nanning, China; licence no. SYXK Gui 2014-0003) were used for all *in vivo* experiments. All animal experiments were performed in accordance with the Guidelines for the Care and Use of Laboratory Animals, and the study protocol was examined and approved by the animal ethics committee of Guangxi Medical University (Nanning, China; approval no. 201809066). A total of 88 animals were randomly divided into six groups: (1) the sham-operated group (sham, *n* = 8) included healthy rats that underwent the sham procedure without CA/CPR, (2) the control saline CA group (CA, *n* = 16) included rats that underwent CA for 7 min and received a transfemoral vein injection of saline after return of spontaneous circulation (ROSC), (3) the 10% glycerol group (Gly, *n* = 16) was used as the vehicle control group, (4) the low-dose PPO group (PPO-L, 10 mg/kg, *n* = 16), (5) the middle-dose PPO group (PPO-M, 20 mg/kg, *n* = 16) and (6) the high-dose PPO group (PPO-H, 40 mg/kg, *n* = 16). One millilitre of PPO weighted to 0.8 g. Due to the rat weight and equal volume injection volume, we dissolved different amount of PPO in 10% glycerine (v/v) to gain three different drug concentrations. All groups, except the sham group, were subjected to 7 min of CA followed by CPR. An equal drug volume was administered intravenously within 10 min after ROSC.

### Experimental CA/CPR model

All rats were starved for 12 h before surgery; however, they had access to water *ad libitum*. To establish the CA/CPR model, rats were anaesthetized with an intraperitoneal injection of 2% pentobarbital sodium (30 mg/kg), as previously described by Chen et al. (2007) and Zheng et al. (2019). Two 20-gauge catheters filled with saline containing 5 IU/mL of sodium heparin were inserted into the left femoral arteriovenous system for hemodynamic monitoring and drug delivery. Pressure transducers were connected to a four-channel physiological recorder (BL-420 E Bio-Systems, Chengdu Technology & Market Co. Ltd., Chengdu, China). After 5 min baseline ECG and physiologic measurements, CA was induced by alternating current AC (12 V) from a stimulator (Chengdu Technology & Market Co. Ltd., Chengdu, China) with transoesophageal pacing electrodes. CA was defined as a loss of aortic pulsation or mean arterial pressure (MAP) <10 mmHg. After endotracheal intubation and 7 min of untreated CA, CPR was initiated with effective ventilation (TV 8 mL/kg, respiration rate 40/min and a positive end-expiratory pressure of 0 cm H_2_O) using a volume-controlled small animal ventilator (DH-150, The Medical Instrument Department of Zhejiang University, Hangzhou, China), oxygenation (100% O_2_) and epinephrine (0.02 mg/kg, IV). Manual chest compressions (180 per min) were controlled following the pace of a metronome, with a depth of 25–30% of the anteroposterior diameter of the animal's thorax and equal compression–relaxation duration by the same investigator. ROSC was defined as an unassisted pulse with a MAP of ≥50 mmHg for ≥1 min. Mechanical ventilation was withdrawn when autonomous respiration recovered up to ≥40 breaths per minute for 1 h after ROSC and the blood pressure stabilized or increased gradually.

### Evaluation of survival rate and neurological deficits

Survival rate and neurological deficits were evaluated 24 h after ROSC. The neurological deficit score (NDS), which includes arousal, reflex, motor, sensory and balance responses, was recorded on a scale of 0–80: 0 corresponds to brain death and 80 corresponds to normal functioning (Geocadin et al. 2000; Jia et al. 2008). Subsequent tests, to evaluate the presence of cerebral injury, were performed on all experimental rats.

### Preparation of brain tissues

Transmission electron microscopy (TEM), immunohistochemical and immunofluorescence staining were conducted on brain tissue (*n* = 3 rats per group). According to AVMA Guidelines for the Euthanasia of Animals: 2013 Edition, rats were injected with 2% pentobarbital sodium (90 mg/kg), causing rodents to lose consciousness. After that, the rats were perfused with normal saline and 4% paraformaldehyde through the aorta. After cerebral perfusion, 50 mg of tissue from the left forehead cortex from each group of rats was placed in 2.5% glutaraldehyde and stored at 4 °C for 24 h for TEM. The remaining brain tissue was fixed in 10% paraformaldehyde and embedded in paraffin. The paraffin blocks were then cut into 3 μm thick sections for haematoxylin and eosin (HE) staining, immunohistochemistry and immunofluorescence, and 5 μm thick sections for Nissl staining.

### HE and Nissl staining

For HE staining, paraffin sections were dewaxed with xylene and washed with alcohol. The sections were then stained with haematoxylin for 3 min and placed in 1% acid alcohol for 5 min. All samples were washed with running tap water until they turned blue. Finally, the tissue samples were observed under an optical microscope after HE staining.

For Nissl staining, the paraffin sections were also dewaxed and stained with Toluidine blue solution (G3668; Solarbio, Beijing, China). They were then stained with 0.5% eosin for 3 s and washed under flowing water. Finally, the Nissl bodies were identified under a light optical microscope and counted from random sections over five different regions in each experimental group using ImageJ software (National Institutes of Health, Bethesda, MD).

### Transmission electron microscopy

Briefly, the samples that were stored in 2.5% glutaraldehyde and were then fixed with 1% osmium tetroxide, dehydrated and embedded in epoxy resin. According to the standard principle of three-dimensional localization, we randomly cut the samples into 100 nm sections. The sections were subsequently fixed in a copper net and double-stained with lead citrate and uranyl acetate. Finally, neuronal morphology was observed and recorded using a Hitachi H-7650 TEM (Shiga, Japan).

### Immunohistochemical and immunofluorescence staining

TNF-α and phospho-MLKL (p-MLKL) immunoreactivity was detected using immunohistochemical staining. First, dewaxed paraffin sections underwent antigen retrieval for 15 min and endogenous peroxidase blocking (ZSGB) for 10 min. The sections were then treated with a normal goat serum blocking solution (ZSGB) for 10–15 min, and subsequently stained with primary antibodies against TNF-α (ab6671, 1:1000; Abcam, Cambridge, UK) or p-MLKL (AF7420, 1:1000; Affinity, Orem, UT) overnight, at 4 °C. The following day, the sections were incubated with goat anti-rabbit IgG polymer (ZSGB) at 25 °C for 20 min. They were then incubated with horseradish enzyme-labelled streptomycin working solution (ZSGB) and counterstained with haematoxylin. The stained sections were observed and interpreted by qualified pathologists under an optical microscope and analysed using ImageJ software (National Institutes of Health, Bethesda, MD).

RIPK3 immunoreactivity was detected using immunofluorescence staining. After the slides were dewaxed, repaired and sealed, the sections were incubated overnight at 4 °C with monoclonal antibodies against RIPK3 (1:3000; Abcam, Cambridge, UK). Tissues were covered with horseradish peroxidase (HRP)-labeled goat anti-rabbit secondary antibody (GB23301, 1:500; Servicebio, Wuhan, China) and incubated for 50 min at room temperature. The slides were then washed thrice with phosphate-buffered solution (PBS; pH 7.4), and CY3 reagent was added for 10 min at room temperature. After incubation, the slides were slightly dampened and dried, and autofluorescence was quenched for 5 min. They were then washed under running water for 10 min. Sections were incubated in 4′,6-diamidino-2-phenylindole (DAPI) solution at room temperature in darkness for 10 min. After washing with PBS, the cover slides were sealed with an autofluorescence quenching sealing agent (G1401; Servicebio, Wuhan, China). The excitation wavelength of CY3 was 510–560 nm, and the emission wavelength was 590 nm (corresponding to red-coloured light). The slides were observed under an inverted fluorescence microscope (Olympus, Tokyo, Japan) and images were collected.

### Western blotting

Western blotting analysis was performed with proteins that were extracted from brain tissues (*n* = 5 rats per group). Briefly, the extracted proteins were centrifuged at 13,000×*g* for 15 min at 4 °C to remove debris followed by evaluation using a BCA protein assay kit (P0012, Beyotime, Shanghai, China) to test protein concentration. The protein was then resolved using sodium dodecyl sulphate-polyacrylamide gel electrophoresis and subsequently transferred onto a poly-vinylidene difluoride membrane (PVDF; ISEQ00010, pore size 0.2 μm; EMd Millipore, Billerica, MA). The membrane was then incubated overnight at 4 °C with rabbit anti-rat primary antibodies against TNF-α (ab6671, 1:1000; Abcam, Cambridge, UK), TNFR1 (ab19139, 1:1000; Abcam, Cambridge, UK), RIPK3 (ab62344, 1:1000; Abcam, Cambridge, UK), MLKL (PA5-43960, 1:1000; Thermo Fisher, Waltham, MA), p-MLKL (Ser358) (AF7420, 1:1000; Affinity, Orem, UT), β-tubulin (ab6046, 1:1000; Abcam, Cambridge, UK) and mouse anti-RIPK1 (ab72139, 1:500; Abcam, Cambridge, UK). The following day, the sections were incubated with anti-rabbit/mouse HRP-conjugated secondary antibodies (1:10,000, Santa Cruz, Dallas, TX). Finally, images were collected using a Gene Genius chemiluminescent detection system (Syngene, Frederick, MD) after enhanced chemiluminescence (ECL). Each protein band was quantified using ImageJ software version 1.46 (National Institutes of Health, Bethesda, MD) and normalized to the expression of β-tubulin.

### Statistical analysis

GraphPad Prism 6.02 software for Windows (GraphPad Software Inc., La Jolla, CA) was used for all statistical analyses. Data are expressed as the mean ± standard error of the mean (SEM), and the assumptions of normality were assessed using the Shapiro–Wilk test. A one-way analysis of variance (ANOVA) was used for comparisons between groups, and pairwise comparisons were conducted using Tukey’s *post hoc* tests when applicable. *p* < 0.05, *p* < 0.01 and *p* < 0.001 were all considered statistically significant.

## Results

### Gas chromatography–mass spectrometry analysis of pomelo peel oil

**Figure 1.**
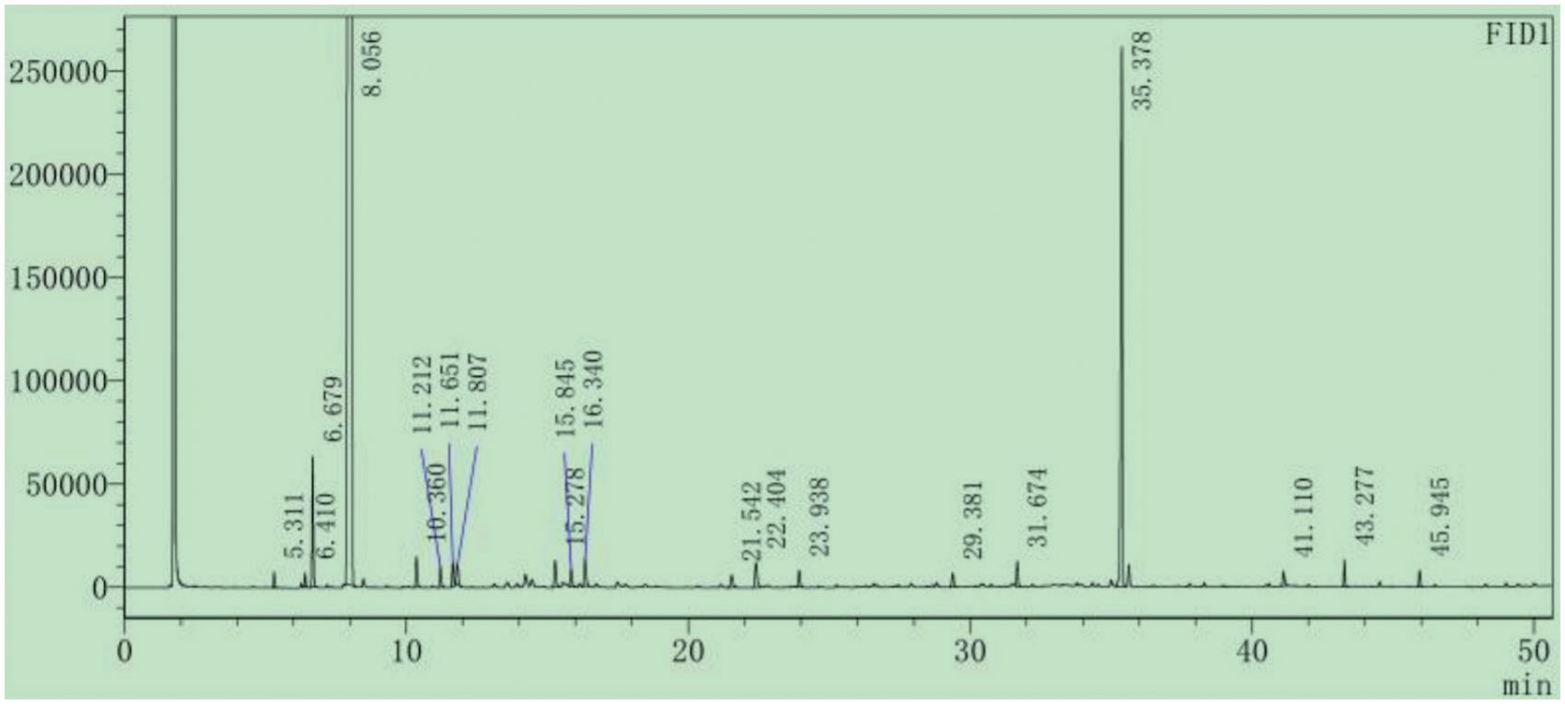
GC–MS chromatogram of PPO. GC–MS: gas chromatography–mass spectrometry; PPO: pomelo [*Citrus maxima* (Burm.) Merr. *cv. Shatian Yu*] peel oil.

The corresponding chromatographic and mass spectrometric data were obtained by GC–MS, and the mass spectra of the peaks in the total ion flow diagram were scanned by mass spectrometry. The mass spectra of the separated compounds were searched for using the National Institute of Standards and Technology database. The chemical components of the analytical samples were determined and combined with the related literature, and the relative percentage content of each component was determined using the peak area normalization method. The GC–MS chromatogram of extracted oil from pomelo peel is shown in Figure 1. The PPO organic compounds are shown in Table 1.

**Table 1.** Chemical composition and contents of GC–MS analysis of pomelo [*Citrus maxima* (Burm.) Merr. *cv. Shatian Yu*] peel oil (PPO).

No.	Compound	Molecular formula	Retention time	Peak area (%)
1	α-Pinene	C10H16	5.311	0.109
2	β-Pinene	C10H16	6.410	0.103
3	Myrcene	C10H16	6.679	1.030
4	Limonene	C10H16	8.056	89.748
5	γ-Terpinene	C10H16	10.360	0.316
6	Limonene oxide	C10H16O	11.212–11.807	0.72
7	α-Terpineol	C10H18O	15.278	0.319
8	L-Carvone	C10H14O	15.845	0.210
9	Caryophyllene	C15H24	16.340	0.321
10	α-Caryophyllene	C15H24	21.542	0.174
11	α-Gurjunene	C15H24	22.404	0.344
12	8-Cedren-13-ol	C_15_H_24_O	23.938	0.186
13	Nerolidol	C15H26O	29.381	0.154
14	Globulol	C15H26O	31.674	0.243
15	Nootkatone	C15H22O	35.378	5.526
16	Osthole	C15H16O3	41.110	0.168
17	Eicosane	C20H42	43.277	0.186
	Total			99.857

A total of 17 compounds were identified by GC–MS analysis of PPO (Table 1). The detected compounds accounted for 99.857% of the total sample. The total ion chromatogram (TIC) is shown in Figure 1. The most abundant compounds identified in PPO were terpenoids. PPO also contained small quantities of alcohols and alkanes. The main constituents in descending order of content were limonene (89.748%), nootkatone (5.526%), myrcene (1.03%), limonene oxide (0.72%), α-gurjunene (0.344%), caryophyllene (0.321%), α-terpineol (0.319%) and γ-terpinene (0.316%). In addition, PPO contained other terpenoids, steroids and eicosane (Table 1).

### Survival rate, CPR duration, NDS changes, heart rates and mean arterial pressure in each group

There was no significant statistical difference in CPR duration between the groups (Table 2). In the sham group, there was a survival rate of 100% and the NDS was 80. Additionally, the survival rate of rats in the CA, Gly and PPO-L groups was lower than that of rats in the sham group. Additionally, the NDS of the CA (NDS = 70), Gly (NDS = 71) and PPO-L (72) groups was lower than the NDS in the sham group (*p* < 0.001 for all comparisons). Additionally, the survival rates and NDS of the PPO-M and PPO-H groups were higher than that of the CA group (*p* < 0.01 for PPO-M and *p* < 0.05 for PPO-H). There was no significant difference in the NDS between the CA and Gly groups (Table 2). Otherwise, there were no significant differences between the groups in the heart rate (HR) or MAP during baseline, CA or ROSC (*p* > 0.05) (Table 3).

**Table 2.** Survival rate, cardiopulmonary resuscitation duration and neurological deficit score in rats at 24 h after cardiopulmonary resuscitation.

Group	Survival rate, *n* (%)	*n*	CPR duration (s), mean ± SEM	NDS, mean (min, max)
Sham	8/8 (100%)	8	–	80 (80, 80)
CA	9/16 (56.25%)	9	90.78 ± 7.73	70 (65, 73)***
Gly	10/16 (62.5%)	10	93.8 ± 10.62	71 (68, 74)***
PPO-L	12/16 (75%)	12	97.58 ± 9.83	72 (70, 77)***
PPO-M	14/16 (87.5%)	14	96.29 ± 10.31	75 (72, 78)^##,^^&^
PPO-H	13/16 (81.25%)	13	92.08 ± 10.73	74 (70, 78)^#^

CA: cardiac arrest/0.9% saline group; CPR: cardiopulmonary resuscitation; Gly: 10% glycerol group; H: high; L: low; M: middle; NDS: neurological deficit score; PPO: pomelo peel oil.

*** *p*< 0.001 vs. sham.

^#^*p*< 0.05 and ^##^*p*< 0.01 vs. CA.

& *p*< 0.05 vs. Gly.

**Table 3.** Heart rates (HRs) and mean arterial pressure (MAP) at baseline, cardiac arrest (CA) or restoration of spontaneous circulation (ROSC) in survival rats.

Group	*n*	HR (beats/min)				MAP (mmHg)	
		baseline	CA	ROSC	baseline	CA	ROSC
Sham	8	348.3 ± 18.12	–	–	79.77 ± 2.44	–	–
CA	9	357.3 ± 19.1	17 ± 5.47	333.2 ± 17.87	81.54 ± 4.51	0.88 ± 0.28	77.14 ± 5.43
Gly	10	357.3 ± 14.86	19.2 ± 4.37	328.5 ± 9.72	88.32 ± 5.54	1.64 ± 0.49	76.83 ± 5.32
PPO-L	12	369.9 ± 12.71	16.92 ± 4.28	372.7 ± 17.36	71.54 ± 1.91	0.73 ± 0.19	71.95 ± 3.92
PPO-M	14	360.4 ± 18.65	14.79 ± 2.92	323.5 ± 14.21	77.68 ± 3.44	0.7 ± 0.22	78.12 ± 4.56
PPO-H	13	344.3 ± 11.81	16.38 ± 3.44	329.8 ± 17.5	82.95 ± 5.15	0.54 ± 0.14	80.25 ± 3.27

HRs: heart rates; MAP: mean arterial pressure; ROSC: restoration of spontaneous circulation; CA: cardiac arrest/0.9% saline group; Gly: 10% glycerol group; H: high; L: low; M: middle; NDS: neurological deficit score; PPO: pomelo peel oil.

### Neuron morphological changes in groups

**Figure 2.**
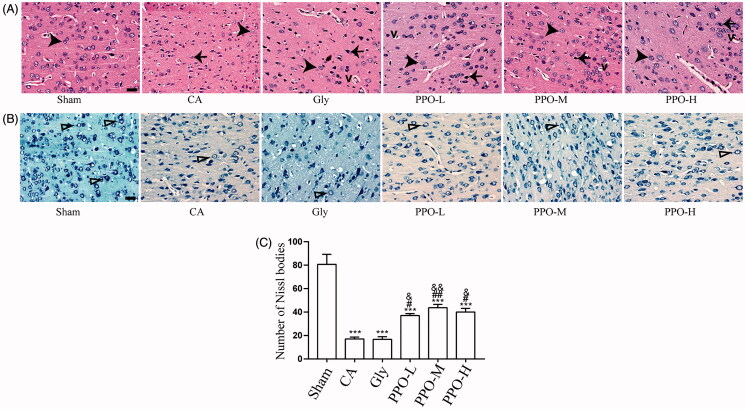
HE and Nissl staining of the rat cerebral cortex. (A) HE staining of the cerebral cortex after CA/CPR for 24 h showing normal morphology of neuronal cells (large arrowheads), necrotic cells (arrows) and vacuolar cells (v). Scale bar: 50 μm. (B) Nissl staining of the cerebral cortex after CA/CPR for 24 h showing Nissl bodies (i.e., surviving neuronal cells) (triangular arrows). Scale bar: 50 μm. (C) Number of Nissl bodies per group. All data are presented as the mean ± SEM. ***p< 0.001 vs. sham; ^#^*p*< 0.05 and ^##^*p*< 0.01 vs. CA; ^&^*p*< 0.05 and ^&&^*p*< 0.01 vs. Gly. HE: haematoxylin and eosin; CA: cardiac arrest/0.9% saline group; CPR: cardiopulmonary resuscitation; SEM: standard error of the mean; sham: sham-operated group; Gly: 10% glycerol group.

Results of HE staining revealed that the neurons in the sham group were intact and round and exhibited normal fibre structures (Figure 2(A)). Additionally, the arrangement of neurons in the cortical areas of rats in the CA and Gly groups were loose as compared with that in the sham group. Additionally, we observed necrotic cells, nuclear deformation and pyknosis, cell oedema and vacuoles in the CA and Gly groups. Further, the number of necrotic cells observed in all of the PPO groups as decreased as compared with that in the CA and Gly groups. The PPO-M group showed the best phenotype, and only a small number of necrotic neurons and vacuoles were observed (Figure 2(A)).

We observed a large number of light-blue Nissl bodies in the cytoplasm of neuronal cells in the sham group (Figure 2(B)). The number of Nissl bodies was decreased in the CA and Gly groups as compared to that in the sham group, and this was due to chromatolysis (*p* < 0.001). However, the number of Nissl bodies was found to be increased in all of the PPO groups as compared with that in the CA and Gly groups (*p* < 0.05 for PPO-L; *p* < 0.01 for PPO-M; and *p* < 0.05 for PPO-H) (Figure 2(C)).

**Figure 3.**
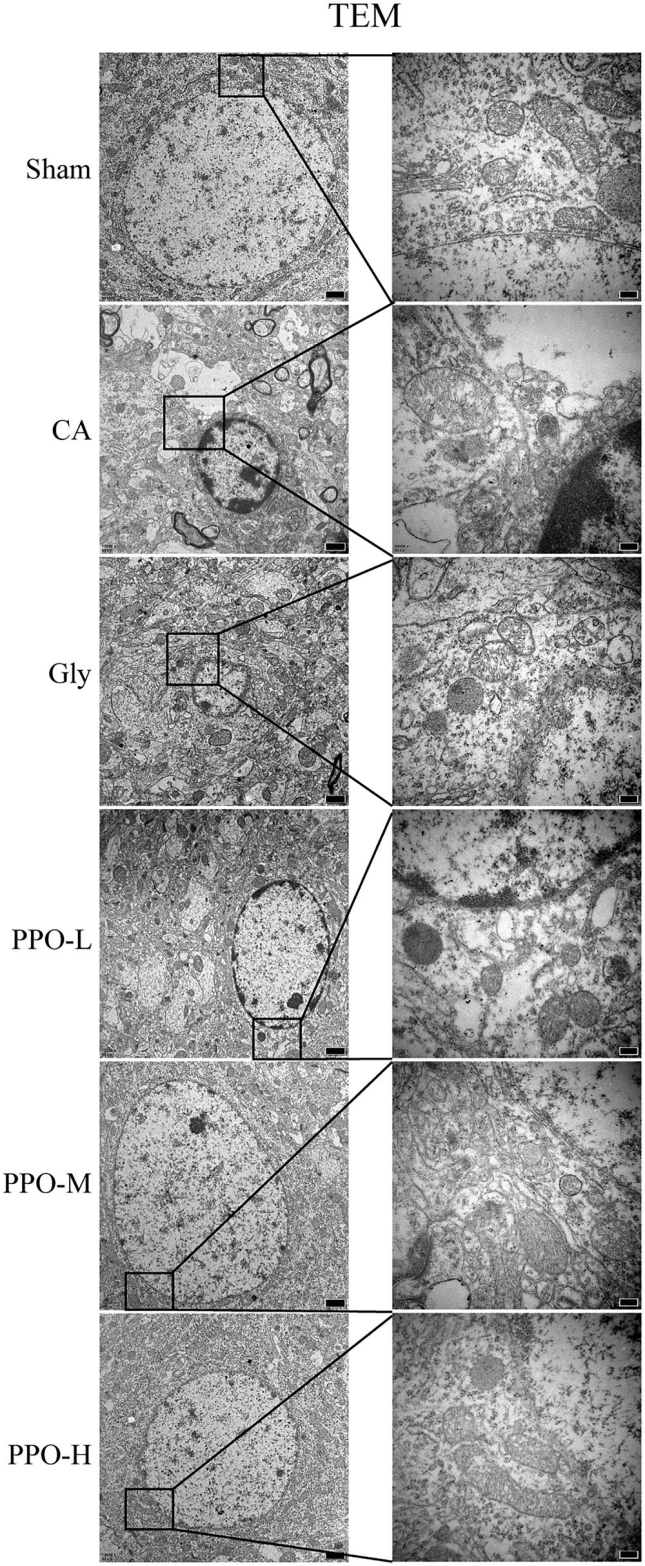
TEM of the rat cerebral cortex at 24 h after CA/CPR. Neuronal cells showing necroptosis, organelle swelling, mitochondrial crest rupture or loss, and plasma membrane discontinuation. Scale bar in the left panels: 1 μm; scale bar in the right panels: 200 nm. CA: cardiac arrest/0.9% saline group; CPR: cardiopulmonary resuscitation.

Results from TEM revealed ultrastructural changes in the characteristics of necroptosis in all rats that underwent CA/CPR. At 24 h after CA/CPR, the neurons of rats that underwent CA/CPR showed irregular endoplasmic reticulum, follicles and swollen mitochondria with ruptured or inexistent cristae. Furthermore, the cell membranes were discontinuous, nuclei were deformed and chromatin was concentrated. These changes were lighter in the PPO groups than those in the CA and Gly groups (Figure 3).

### PPO can reduce TNF-α, p-MLKL and RIPK3 immunoreactivity in the cortex

**Figure 4.**
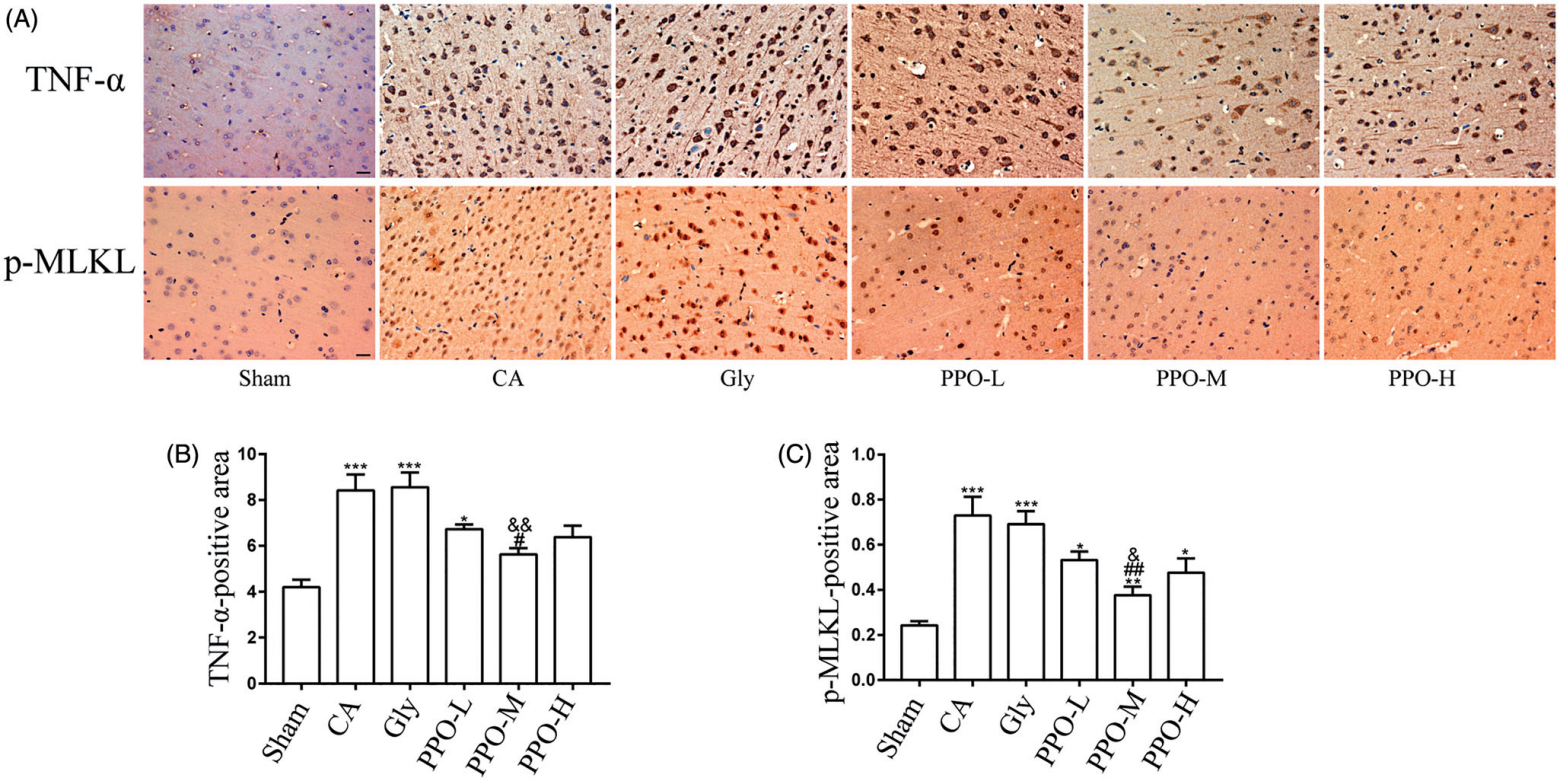
Immunohistochemistry for TNF-α and p-MLKL. (A) Immunohistochemical staining of TNF-α and p-MLKL in the cerebral cortex after CA/CPR for 24 h. The dark granules detected in the cytoplasm and nucleus of cortical cells were positive for TNF-α and p-MLKL. Scale bar: 20 μm. (B, C) TNF-α- and p-MLKL-positive area per group, respectively. All data are presented as the mean ± SEM. *p < 0.05, **p < 0.01 and ***p < 0.001 vs. sham; ^#^*p* < 0.05 and ^##^*p* < 0.01 vs. CA; ^&^*p* < 0.05 and ^&&^*p* < 0.01 vs. Gly. TNF-α: tumour necrosis factor-α; p-MLKL: phosphorylated mixed lineage kinase domain-like protein; CA: cardiac arrest/0.9% saline group; CPR: cardiopulmonary resuscitation; SEM: standard error of the mean; sham: sham-operated group; Gly: 10% glycerol group.

Results from our immunohistochemistry analyses revealed that the dark granules detected in the cytoplasm and nuclei of cortical cells were TNF-α-and p-MLKL (Figure 4(A)). Additionally, the expression of TNF-α in the CA and Gly groups was significantly higher than that in the sham group (*p* < 0.001). Further, the expression of p-MLKL in all the groups that underwent CA/CPR was higher than that in the sham group (*p* < 0.05). However, the expression levels of TNF-α and p-MLKL were lower in the PPO-M group than those in the CA and Gly groups (*p* < 0.05). There was no significant difference in TNF-α and p-MLKL expression between the CA and Gly groups (*p* > 0.05) (Figure 4(B,C)).

**Figure 5.**
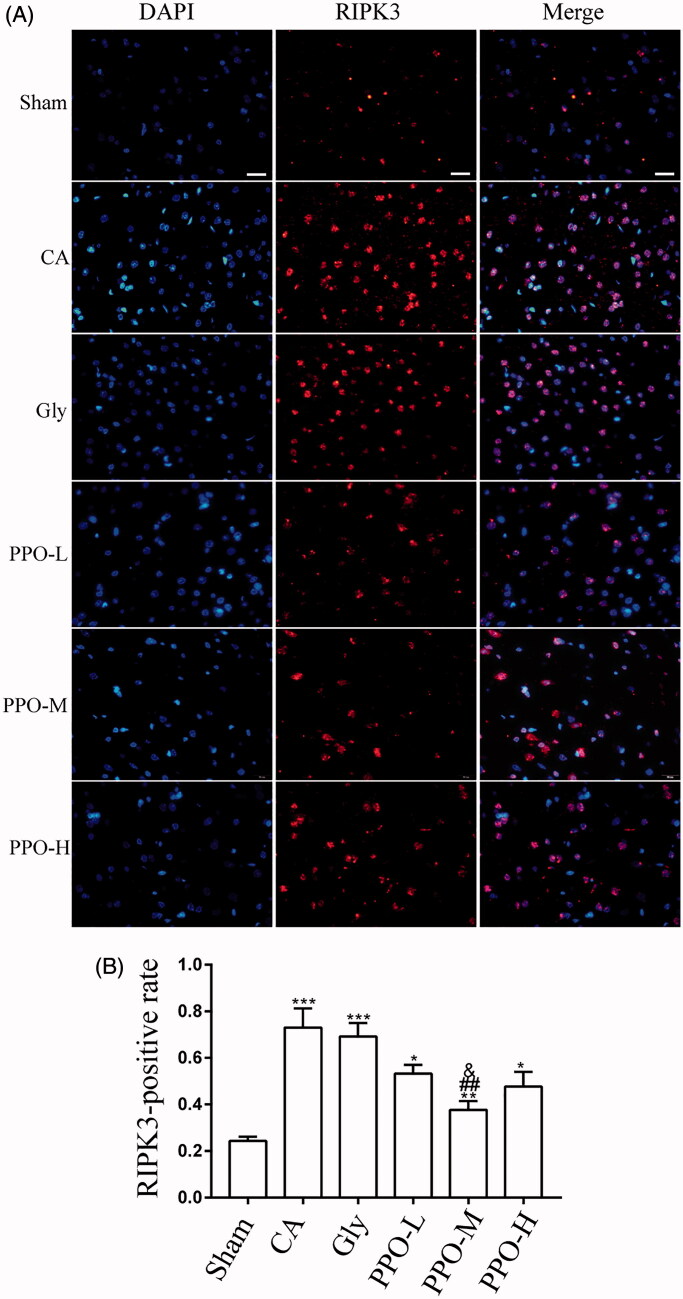
Immunofluorescence for RIPK3. (A) Immunofluorescence staining of RIPK3 in the cerebral cortex at 24 h after CA/CPR. Scale bar: 20 μm. The light color granules detected were positive cells. Scale bar (White short bar): 20 μm. (B) RIPK3-positive rate (RIPK3-positive area/DAPI-positive area) per group. All data are mean ± SEM. *p< 0.05, **p< 0.01 and ***p< 0.001 vs. sham; ^##^*p*< 0.01 vs. CA; and ^&^*p*< 0.05 vs. Gly. RIPK3: receptor-interacting serine/threonine kinase 3; CA: cardiac arrest/0.9% saline group; CPR: cardiopulmonary resuscitation; DAPI: 4′,6-diamidino-2-phenylindole; SEM: standard error of the mean; sham: sham-operated group; Gly: 10% glycerol group.

In order to investigate whether RIPK3, an important mediator of necroptosis, was downregulated after PPO treatment, the level of RIPK3 in the cerebral cortex was determined using immunofluorescence (Figure 5(A)). The RIPK3-positive rate was higher in the groups that underwent CA/CPR than in the sham group (*p* < 0.05). Compared to the CA and Gly groups, the expression of RIPK3 was lower in the PPO-M group (*p* < 0.05) (Figure 5(B)).

### PPO can down-regulate the levels of necroptosis proteins in the cortex

**Figure 6.**
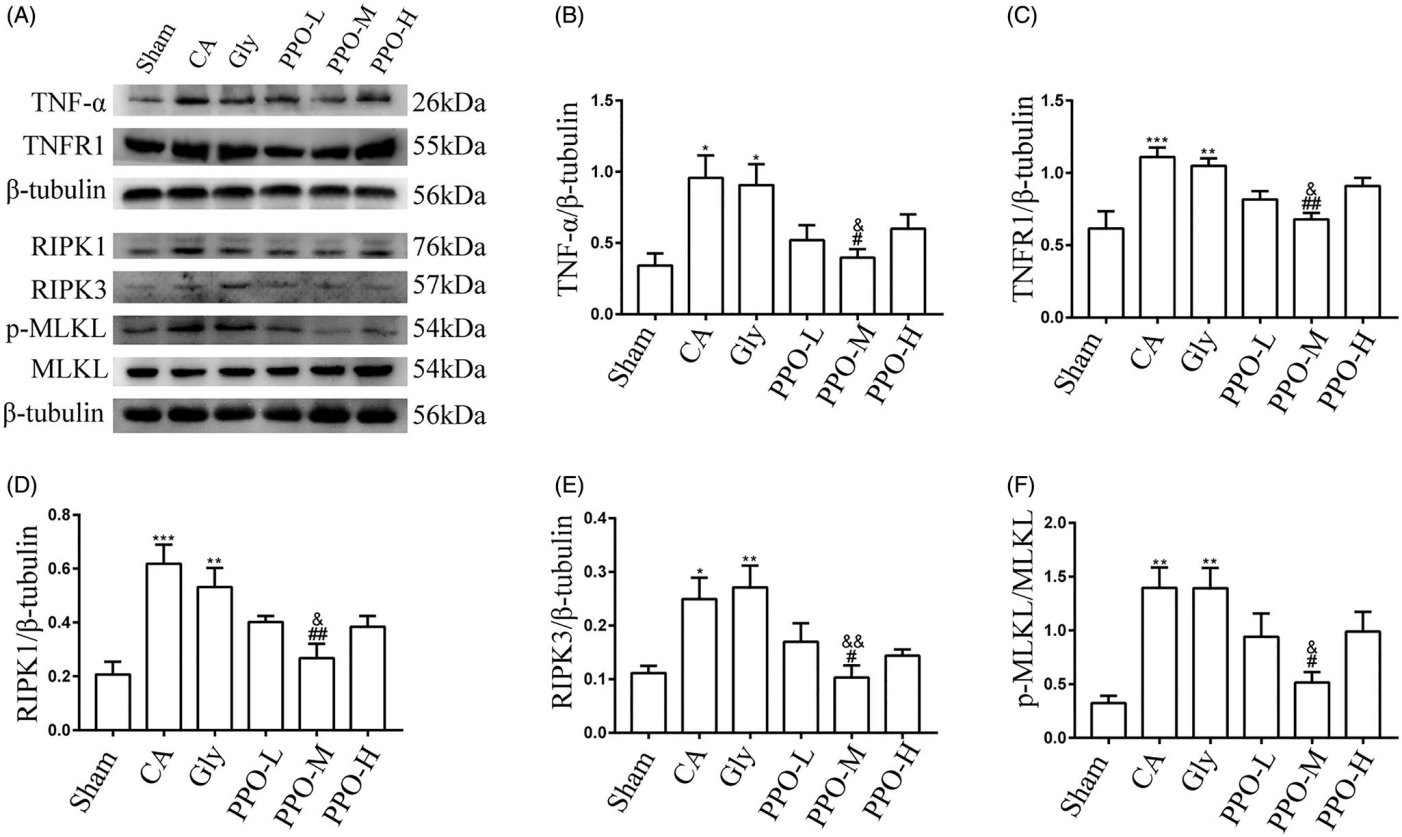
Effect of PPO on the necroptosis signalling pathway. (A) Western blot for necroptosis proteins, including TNF-α, TNFR1, RIPK1/3 and p-MLKL/MLKL. (B–F) Quantification of the western blot protein bands. All data are presented as the mean ± SEM. *p < 0.05, **p < 0.01, and ***p < 0.001 vs. sham; ^#^*p* < 0.05 and ^##^*p* < 0.01 vs. CA; ^&^*p* < 0.05 and ^&&^*p* < 0.01 vs. Gly. PPO: pomelo [*Citrus maxima* (Burm.) Merr. *cv. Shatian Yu*] peel oil; TNF-α: tumour necrosis factor-α; TNFR1: tumour necrosis factor receptor 1; RIPK1/3: receptor-interacting serine/threonine kinase 1/3; MLKL: mixed lineage kinase domain-like protein; p-MLKL: phosphorylated mixed lineage kinase domain-like protein; SEM: standard error of the mean; sham: sham-operated group; CA: cardiac arrest/0.9% saline group; Gly: 10% glycerol group.

In order to explore the effects of PPO on the levels of TNF-α, TNFR1, RIPK1, RIPK3, p-MLKL and MLKL in the cortex, we intravenously injected different concentrations of PPO into rats that underwent CA/CPR. Our results demonstrated that the expression levels of TNF-α, TNFR1, RIPK1, RIPK3, and p-MLKL were significantly increase in the CA and Gly groups as compared with those in the sham group (*p* < 0.05). Additionally, protein expression was lower in the PPO-M group than that in the CA and Gly groups (*p* < 0.05). There was no significant difference in protein expression between the CA and Gly groups (*p* > 0.05) (Figure 6).

## Discussion

**Figure 7.**
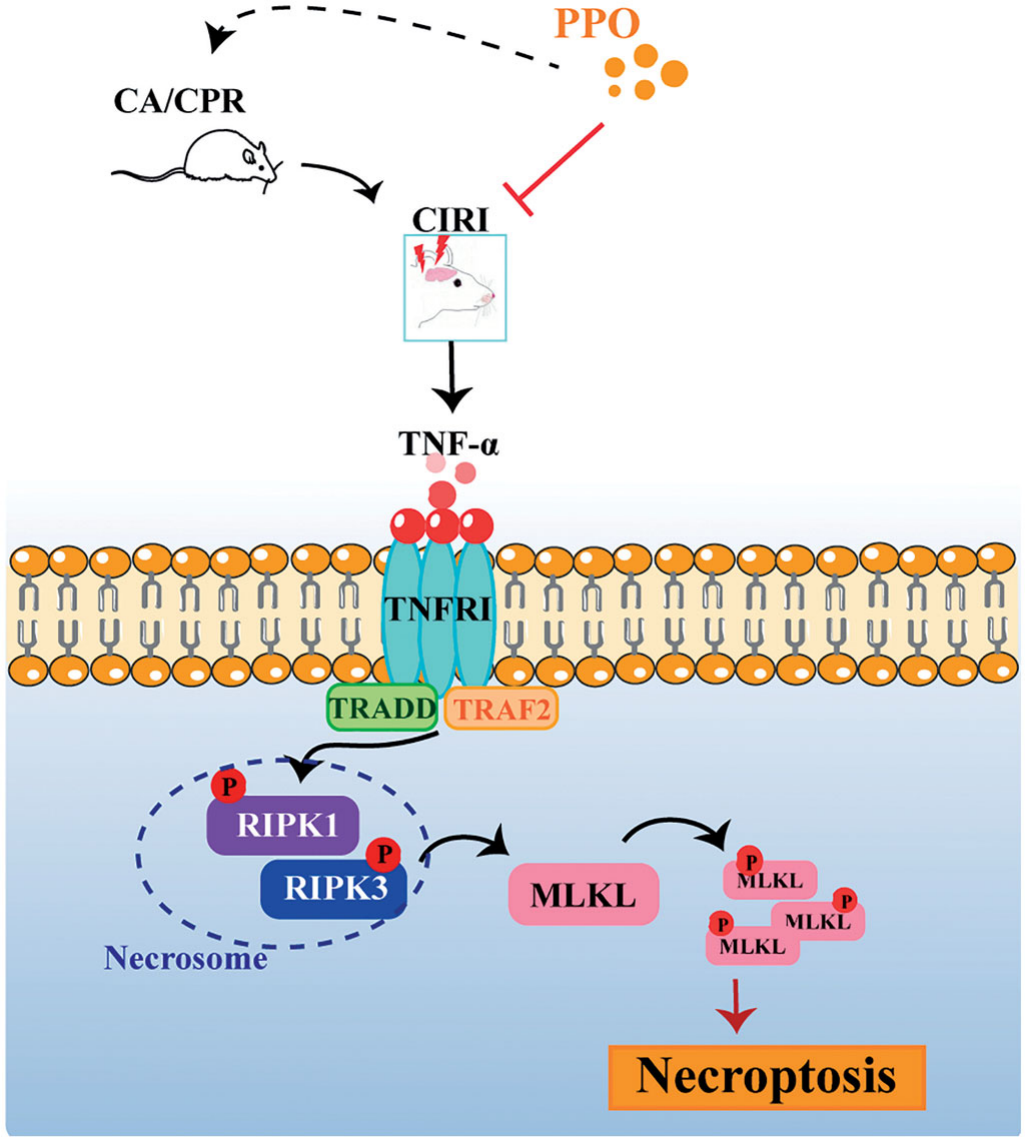
CA/CPR can induce CIRI and activate TNF-α-mediated necroptosis pathways. Pomelo peel oil (PPO) suppressed TNF-α-induced necroptosis and improved CIRI in a rat model of cardiac arrest. CA: cardiac arrest/0.9% saline group; CPR: cardiopulmonary resuscitation; TNF-α: tumour necrosis factor-α; PPO: pomelo [*Citrus maxima* (Burm.) Merr. *cv. Shatian Yu*] peel oil; CIRI: cerebral ischaemia–reperfusion injury.

Multiple studies have attempted to uncover the mechanisms underlying CIRI pathology and identify agents that reduce brain damage (Yang et al. 2019). According to the results of GC–MS analysis, the three main constituents of PPO in descending order were limonene (89.748%), nootkatone (5.526%) and myrcene (1.03%) (Table 1). Due to different plant origin, growth environment and methods of obtaining oil, the proportion of compounds in our sample was different from that reported in the literature (Chen et al. 2018; He et al. 2019). Similarly, the main component of PPO is limonene, and our sample also contained α-pinene (0.109%) and β-pinene (0.103%). In this study, we showed that PPO had a neuroprotective effect against CIRI in a rat model of CA/CPR (Figure 7). In particular, PPO improved neurological deficits and cerebral morphologic structure, and suppressed the expression of necroptosis proteins.

CIRI is related to a series of pathological mechanisms such as oxidative stress, inflammation, apoptosis and necroptosis (Mozaffarian et al. 2015). When cell injury occurs, necroptosis can be induced by death signals, such as TNF-α, toll-like receptors and interferon (Deng et al. 2019). Recently, studies have confirmed that TNF-α is the main trigger of necroptosis (Dhuriya and Sharma 2018). When TNF-α binds to TNFR1, a TNF-α-TNFR1 complex is formed, and TNF receptor-associated death domain (TRADD) is recruited (Fayaz et al. 2014). The death domain (DD) in TRADD combines with the DD of TNFR1, which signals to RIPK1 and RIPK3 independently of cysteine aspartate enzyme-8 (caspase-8). Activated RIPK1/3 proteins induce the expression of p-MLKL. P-MLKL translocates into the inner leaflet of the plasma membrane and disturbs cell integrity, causing cell death (Fauster et al. 2015; Martens et al. 2017). Growing evidence suggests that necroptosis induces cell death in ischaemic brain injury (Yang et al. 2017). RIPK1/RIPK3 reach their highest levels 24 h after CA/reperfusion, and necrosis occurs (Xu et al. 2016; Ryan et al. 2018). Newton et al. (2016) reported that RIPK1 and RIPK3 deficiency inhibited the release of inflammatory factors, such as TNF-α, and reduced neuronal cell death. In our study, as shown in Figure 7, PPO inhibited the expression of TNF-α, TNFR1 and necroptosis proteins, suggesting that PPO exerts its neuroprotective effect by suppressing TNF-α-induced necroptosis.

TNF-α directly induces oxidative stress by activating ROS synthetases and activating nuclear factor κ-light-chain-enhancer of activated B cells (NF-κB) (Fischer and Maier 2015; Ang and Ting 2019). In TNF-mediated necroptosis, RIPK1/RIPK3 is upregulated. The upregulation of RIPK1/RIPK3 is followed by the activation of p-MLKL, production of ROS and aggravation of cell injury (Yang et al. 2018). Recently, studies have reported that oxidative stress during CIRI depends (at least partly) on necroptosis (Li et al. 2020). Although there is no research to prove PPO related to necroptosis, He et al. (2019) reported that PPO showed antioxidant activity against the formation of superoxide anion radicals. Furthermore, many compounds in PPO also have been shown antioxidant and anti-inflammatory neuroprotective effects. Shin et al. (2020) demonstrated that limonene has a neuroprotective function, reducing oxidative stress and inflammation to resist the neurotoxicity of the β-amyloid 42. Although there is no research on the relationship between nootkatone and ischaemic brain injury, there is evidence that it plays a neuroprotective role in a mouse model of Alzheimer's disease through inhibition of the TLR4/NF-κB/NLRP3 pathway and reducing the inflammatory response (Qi et al. 2019). Furthermore, Burcu et al. (2016) confirmed that β-myrcene had neuroprotective effects on oxidative and neuronal injury mediated by global CA/reperfusion in C57BL/J6 mice and α-pinene also had been shown that antioxidant enzymes activity in ischaemic nerve injury (Khoshnazar et al. 2019). In this study, PPO showed a neuroprotective effect against CIRI. Since PPO suppressed the expression of TNF-α and necroptosis mediators, there may be a link between necroptosis and oxidative stress.

In the current study, all three doses of PPO, especially PPO-M, led to improvements in cerebral function and morphology and increased the survival rate of rats at 24 h after CA/CPR. PPO contains a variety of components that have the same or different effective targets; therefore, antagonism may occur. PPO-M may be the best matching concentration for synergism, since it showed stronger effects than PPO-L and PPO-H. Based on this finding, it would be useful to evaluate the plasma levels of specific ingredients of PPO and their effects. Although pomelo is a kind of popular subtropical fruit, pomelo peel is discarded because of unrecognized health benefits. Our research suggests that pomelo peel may be used as a natural health product resource. Further, we will study and compare the effects of the main components of PPO on CIRI.

## Conclusions

Our findings suggest that the necroptosis signalling pathway is a therapeutic potential target in CIRI after CA/CPR. PPO had a neuroprotective effect against CIRI in a rat model of CA/CPR, and these neuroprotective effects may have been achieved via the regulation of TNF-α-induced necroptosis.
